# Zearalenone causes ovarian damage and abnormal estradiol secretion in meat rabbits by inducing oxidative stress and inflammatory responses

**DOI:** 10.3389/fvets.2025.1566284

**Published:** 2025-04-10

**Authors:** Fengyang Wu, Fengxia Wang, Zhaohong Tang, Xinyu Yang, Yanhua Liu, Man Zhao, Shudong Liu, Shuaijuan Han, Baojiang Chen

**Affiliations:** ^1^College of Animal Science and Technology, Hebei Agricultural University, Baoding, China; ^2^College of Food Science and Technology, Hebei Agricultural University, Baoding, China; ^3^Hebei Research Institute of Microbiology Co., Ltd., Baoding, China

**Keywords:** zearalenone, meat rabbit, ovary, oxidative stress, inflammatory response, reproductive hormone

## Abstract

Zearalenone (ZEA), a prevalent mycotoxin in animal feeds, is known to disrupt normal ovarian development and function due to its estrogenic activity. This study investigates the toxic effects of ZEA on the ovaries of meat rabbits and explores the underlying mechanisms. Ninety healthy 41-day-old Hyla male rabbits were randomly assigned into three groups. The control group received a basal diet, while the experimental groups were fed basal diets supplemented with 300 and 600 μg/kg ZEA, respectively. Each group consisted of 30 replicates, with one rabbit per replicate, and the experimental period lasted 42 days. The results showed that, compared to the control group, the ovarian index was significantly increased in the 600 μg/kg ZEA supplementation group (*p* < 0.05). In addition, ovarian tissue exhibited pathological changes, including follicular dilatation, thinning of the follicular granulosa, punctate necrosis of granulosa cells, deep stained cytosolic nuclei, and nuclear fragmentation. Compared to the control group, the 600 μg/kg ZEA supplementation group exhibited significantly elevated blood levels of gonadotropin-releasing hormone, luteinizing hormone, estradiol, malondialdehyde (MDA), and interleukin 1β (IL-1β) (*p* < 0.05). Conversely, total antioxidant power (TAOC) and glutathione peroxidase (GSH-Px) activities were significantly reduced in this group (*p* < 0.05). The level of MDA in the ovarian tissue of rabbits in the 600 μg/kg ZEA supplementation group was significantly elevated compared to the control group, while the activities of GSH-Px and TAOC were significantly reduced (*p* < 0.05). Moreover, the expression levels of luteinizing hormone receptor mRNA, heat shock protein 70 mRNA, tumor necrosis factor-α mRNA, and *IL-1β* mRNA in the ovarian tissue significantly increased, whereas the expression of copper and zinc superoxide dismutase mRNA was significantly decreased compared to the control group (*p* < 0.05). In conclusion, supplementation with 600 μg/kg ZEA induces oxidative stress and inflammatory responses in the ovaries of meat rabbits by modulating the expression of related genes. These effects disrupt ovarian development, cause pathological changes, and impair the secretion of reproductive hormones. This study is the first to report the toxic effects of ZEA on the ovaries of Hyla rabbits and provides preliminary insights into its underlying mechanisms.

## 1 Introduction

Zearalenone (ZEA) is a polyketide secondary metabolite primarily produced by *Fusarium graminearum* (1). It is one of the most prevalent mycotoxins detected in various types of rabbit feeds (2). The heterocyclic phenolic hydroxyl groups in ZEA and its derivatives closely resemble those found in natural estrogens, enabling them to competitively bind to estrogen receptors. This interaction can disrupt the development and function of reproductive organs, resulting in reproductive toxicity (3). According to literature, exposure to ZEA can disrupt the estrous cycle, inhibit the secretion of luteinizing hormone (LH) and estradiol, reduce the number of primary and mature follicles, and ultimately induce ovarian damage in mice (4). Moreover, ZEA exposure has been shown to cause cell cycle arrest in granulosa cells, leading to cellular damage and apoptosis within the ovarian tissue (5). Exposure to ZEA can lower the reproductive ability of female animals by interfering with the neuroendocrine regulatory function of the hypothalamic-pituitary-ovarian axis (HPOA) and inducing abnormal secretion of sex hormones (6). It is a crucial factor contributing to the accelerated atresia of ovarian follicles and the deficiency of luteal function (7, 8). As one of the most harmful mycotoxins in rabbit production, ZEA is not only associated with reproductive toxicity but also exerts organotoxic effects on the liver, kidney, and intestine, in addition to causing immunotoxicity and cytotoxicity (9–11).

One of the mechanisms by which ZEA exerts its toxic effects is through the induction of oxidative stress and damage. Studies have demonstrated that ZEA disrupts the balance between the antioxidant and oxidative systems by causing mitochondrial dysfunction, inducing endoplasmic reticulum stress, and inhibiting the activity of antioxidant enzymes, thereby promoting oxidative stress and damage (9, 12, 13). It has been reported that exposure to ZEA induces mitochondrial damage in mouse liver cells, leading to the overproduction of reactive oxygen species (ROS), which in turn causes oxidative stress and liver damage (12). Additionally, ZEA contributes to injury in chicken embryo fibroblasts by inducing endoplasmic reticulum stress (13). ZEA reduces the activity of antioxidant enzymes and the antioxidant capacity in meat rabbits by inhibiting the expression of some key genes in the nuclear factor E2-related factor 2 (Nrf2)/antioxidant response element (ARE) signaling pathway (9). Furthermore, ZEA can exert toxic effects by triggering inflammatory responses. Many immune cells, including macrophages, B cells, and NK cells, express estrogen receptors on their surface, allowing ZEA to modulate the immune response through estrogen-like activity (14, 15). A previous study reported that exposure to ZEA significantly increased the levels of tumor necrosis factor-α (TNF-α) and interleukin-4 (IL-4) while significantly decreasing interleukin-10 (IL-10) levels in the liver of meat rabbits, leading to inflammatory responses and injuries (9). It is worth noting that the inflammatory response is an important pathway through which ZEA and other environmental estrogens induce apoptosis in ovarian cells and impair ovarian function (16).

The ovary, as a critical reproductive organ in rabbits, is responsible for oocyte production and release, hormone secretion, and other essential physiological functions. It is a target organ for ZEA toxicity, with exposure leading to a range of ovarian disorders, such as granulosa cell damage and apoptosis, reduced ovarian reserve, abnormal ovarian development, and hormone secretion dysfunction, which directly impair the physiological function of the ovary (17–19). This study, for the first time, elucidates the toxic effects of ZEA on the ovaries of Hyla rabbits, along with preliminary insights into the underlying mechanisms involving oxidative stress and inflammatory response. Our findings will provide valuable scientific references for establishing limit standards for ZEA in rabbit production and for the prevention and control of ZEA contamination.

## 2 Materials and methods

### 2.1 Experimental materials and basal diets

ZEA, with ≥98% purity, was purchased from TripleBond Scientific Inc. (Guelph, Canada). The basal diets were formulated and prepared based on the nutrient requirements for rabbits as outlined by the NRC (1977), with their composition and nutrient levels shown in Table 1.

**Table 1:** Composition and nutrient levels of the basal diet (air-dry basis, %).

Ingredients	Content
Wheat bran	33.00
Peanut vite	15.00
Peanut hull	12.50
Soybean meal	11.00
Corn	8.00
Artemsia argyi powder	8.00
Rice bran	5.00
Wheat middling	4.00
Limestone	1.05
NaCl	0.50
Ca(HCO_3_)_2_	0.50
Lysine	0.30
Methionine	0.15
Premix^1^	1.00
Total	100.00
Nutritional level^2^	
Digestible energy, MJ/kg	9.33
Crude protein	16.37
Ether extract	3.40
Crude fibre	14.91
Neutral detergent fibre	42.08
Acid detergent fibre	23.97
Acid detergent lignin	5.62
Crude ash	12.35
Calcium	1.60
Total phosphorus	0.80

1 The premix provided the following per kilogram of diet: Fe: 70 mg; Cu: 20 mg; Zn: 70 mg; Mn: 10 mg; Co: 0.15 mg; I: 0.2 mg; Se: 0.25 mg; vitamin A: 10,000 IU; vitamin B_1_: 2 mg; vitamin B_2_: 6 mg; vitamin B_3_: 50 mg; vitamin B_5_: 50 mg; vitamin B_6_: 2 mg; vitamin B_12_: 0.02 mg; vitamin D: 900 IU; vitamin E: 50 mg; vitamin K: 2 mg; Biotin: 0.2 mg; choline chloride: 1000 mg.

2 The digestible energy of the nutritional level were calculated, while the others were measured.

Mycotoxin levels (ZEA, aflatoxin B1, and vomitoxin) were measured after the diet preparation using ELISA kits. The ZEA contents in each group were 63.91, 351.08, and 675.32 μg/kg, respectively, while aflatoxin B1 and vomitoxin were undetectable in the diets of all groups. The ELISA kits were purchased from Shenzhen Finder Biotech Co., Ltd. (Shenzhen, China).

### 2.2 Experimental grouping and feeding management

Ninety healthy Hyla rabbits with similar body weights (1.35 ± 0.08 kg) were randomly divided into three groups, with 30 replicates per group and one rabbit per replicate. The rabbits in the three groups were fed basal diets supplemented with 0, 300, and 600 μg/kg ZEA, respectively. The rabbits were housed in individual cages with ad libitum access to food and water for 42 days.

The experimental protocols were approved by the Animal Care and Use Committee of Hebei Agriculture University (Baoding, China) (Protocol: 2023139).

All animal experiments complied with the ARRIVE guidelines were carried out in accordance with the U.K. Animals (Scientific Procedures) Act, 1986 and associated guidelines, EU Directive 2010/63/EU for animal experiments.

### 2.3 Measurement indicators and methods

#### 2.3.1 Growth performance and ovarian index

The fasting body weight of rabbits in each group was measured at the beginning and end of the experiment, and the average daily gain (ADG) was calculated. The average daily feed intake (ADFI) was determined by weighing the feed provided to each group, and the feed-to-gain ratio (F:G) was calculated based on ADFI and ADG. The live weight of the rabbits was measured before slaughter. Subsequently, the weight of the ovaries was measured after slaughter to calculate the ovarian index using the following formula:

$\begin{array}{l} \mathrm{Ovarian index}\;\left(g/\mathrm{kg}\right)\\ =\mathrm{ovarian weight}\;\left(g\right)/\mathrm{rabbit live weight before slaughter}\;\left(\mathrm{kg}\right)\text{.} \end{array}$

#### 2.3.2 Morphology and structure of ovarian tissue

Ovarian tissue samples were fixed in a 4% paraformaldehyde solution. Paraffin embedding and hematoxylin-eosin staining (HE) staining of the ovarian tissues were performed following the method reported by Wu et al. (9). A microscope camera system (BA200 Digital) was employed for tissue observation and image acquisition (Motic, Xiamen, China).

#### 2.3.3 Serum and ovarian tissue indicators

At the end of the experimental period, eight rabbits with body weights close to the group average were selected from each group for blood collection via cardiac puncture after 12 h of fasting. Blood samples were collected into vacuum tubes and centrifuged at 3,000 × g for 10 min at 4°C to separate the serum. After blood sampling, the rabbits were sacrificed by manual cervical dislocation, and ovarian tissue samples were collected. Both serum and ovarian samples were stored at −80°C for subsequent analysis. The activity of superoxide dismutase (SOD) and the levels of TNF-α, interleukin-1β (IL-1β), IL-4, interleukin-6 (IL-6), IL-10, gonadotropin-releasing hormone (GnRH), follicle-stimulating hormone (FSH), LH, estradiol, and progesterone were measured using ELISA kits obtained from Jiangsu Meimian Biotechnology Co., Ltd. (Yancheng, Chian) and Shanghai COIBO Biotechnology Co., Ltd. (Shanghai, China). The activities of TAOC and GSH-Px were measured using a colorimetric method, while CAT activity was determined via visible light detection. Furthermore, the MDA content was assessed using thiobarbituric acid method. All assay kits were purchased from Nanjing Jiancheng Bioengineering Institute (Nanjing, China).

#### 2.3.4 Expression levels of ovary-related genes

Ovarian tissue samples (100 mg) were collected, and the relative expression levels of follicle-stimulating hormone receptor (*FSHR*), luteinizing hormone receptor (*LHR*), heat shock protein 70 (*HSP70*), *GSH-Px*, copper and zinc superoxide dismutase (*Cu/Zn-SOD*), manganese superoxide dismutase (*Mn-SOD*), *TNF-α*, *IL-1β*, *IL-4*, *IL-6*, *IL-10*, and glyceraldehyde-3-phosphate dehydrogenase (*GAPDH*) were analyzed using qRT-PCR. Primers were designed and synthesized based on gene sequences available in GenBank (Table 2). Total RNA was extracted using a Trizol kit following the manufacturer’s instructions (Invitrogen, Massachusetts, United States), and RNA concentration was measured using a NanoDrop Lite spectrophotometer (Thermo, Massachusetts, United States). Next, cDNA was synthesized using a reverse transcription kit according to the provided protocol (Vazyme, Nanjing, China). qRT-PCR premixes were prepared using a qRT-PCR kit (Vazyme, Nanjing, China), quantitative primers, and cDNA templates. GAPDH was used as the internal reference gene, and all the experiments were performed in triplicate. The expression levels of the target genes were calculated using the $2^{-\mathrm{\Delta \Delta CT}}$ method.

**Table 2:** Sequence of primers for real-time PCR.

Genes	Primer sequence (5′ → 3′)	Accession No.
*FSHR*	Forward: CAGCTCCGAGAGTCACCAAT	XM_002709718.2
	Reversed: CACCTTACTGGTATGCCGGG	
*LHR*	Forward: TGCTGTAAACATCGGGCTGA	S57793.1
	Reversed: GGTGGACTGCACAGGCTTAT	
*HSP70*	Forward: GAGTGAGGAGAGGCGTCAGT	NC_013671.1
	Reversed: GTTCTCACACAGGTCGGACA	
*GSH-Px*	Forward: GCCCAGTCTGTGTACTCCTT	NM_001085444.1
	Reversed: CGTTCTCCTGATGCCCAAAC	
*Cu/Zn-SOD*	Forward: GCAGGCCCTCACTTTAATCC	NM_001082627.2
	Reversed: CCTTTGCCCAAGTCGTCTTC	
*Mn-SOD*	Forward: TGACGGCTGTGTCTGTTGGT	L28808.1
	Reversed: GCAGGTAGTAAGCGTGTTCCC	
*TNF-α*	Forward: CTGCACTTCAGGGTGATCG	NM_001082263.1
	Reversed: CTACGTGGGCTAGAGGCTTG	
*IL-1β*	Forward: CCCCAACCGTTACCCAAAGA	NM_001082201.1
	Reversed: GGGAACTGGGCAGACTCAAA	
*IL-4*	Forward: CCCAAGAACACAACCGAGAG	NM_001163177.1
	Reversed: AGTCTGTCTGGCTTCCTTCC	
*IL-6*	Forward: TCCAGGAGCCCGACTATGAA	NM_001082064.2
	Reversed: TCGTCACTCCTGAACTTGGC	
*IL-10*	Forward: AGAACCACAGTCCAGCCATC	NM_001082045.1
	Reversed: GCTTTGTAGACGCCTTCCTC	
*GAPDH*	Forward: TGCCACCCACTCCTCTACCTTCG	NM_001082253
	Reversed: CGAAGGTAGGGATGGGTGGCA	

*FSHR, follicle stimulating hormone receptor; LHR, luteinizing hormone receptor; HSP70, heat shock protein70; GSH-Px, glutathione peroxidase; Cu/Zn-SOD, copper and zinc superoxide dismutase; Mn-SOD, manganese superoxide dismutase; TNF-α, tumor necrosis factor-α; IL-1β, interleukin-1β; IL-4, interleukin-4; IL-6, interleukin-6; IL-10, interleukin-10; GAPDH, glyceraldehyde-3-phosphate dehydrogenase.*

### 2.4 Statistical analysis

Excel 2021 and SPSS 26.0 were used for the statistical analysis of the data. One-way analysis of variance (ANOVA) was used to test for significant differences between the groups, and Duncan’s method was employed for multiple comparisons. A *p*-value less than 0.05 (*p* < 0.05) was considered indicative of a significant difference.

## 3 Results

### 3.1 Growth performance and ovarian index

The ovarian index of the group supplemented with 600 μg/kg ZEA was significantly higher than that of the control group (*p* < 0.05) (Table 3). There were no significant differences in initial body weight, final body weight, ADG, ADFI, and F/G among all groups (*p* > 0.05).

**Table 3:** Effect of zearalenone-contaminated diet on the growth performance and ovarian index of meat rabbits.

Items	Control group	300 μg/kg ZEA group	600 μg/kg ZEA group	SEM	*p*-value
Initial body weight, g	1340.83	1355.17	1352.50	18.91	0.953
Final body weight, g	3098.60	3081.65	3105.23	75.58	0.992
ADG, g	41.85	41.11	41.73	1.226	0.968
ADFI, g	177.14	171.94	179.25	4.026	0.739
F/G	4.32	4.29	4.34	0.090	0.972
Ovarian index, g/kg	0.07^a^	0.08^ab^	0.12^b^	0.010	0.042

^a–c^Within a row, means with different superscripts differ significantly (*p* < 0.05). Values are means (initial body weight, final body weight, ADG, ADFI, F/G, *n* = 30; ovarian index, *n* = 8). 300 μg/kg ZEA, group, 300 μg/kg zearalenone supplementation group; 600 μg/kg ZEA, group, 600 μg/kg zearalenone supplementation group; SEM, the standard error of the means; ADG, average daily gain; ADFI, average daily feed intake; F:G, feed-to-gain ratio.

### 3.2 Structure and morphology of ovarian tissue

**Figure 1:**
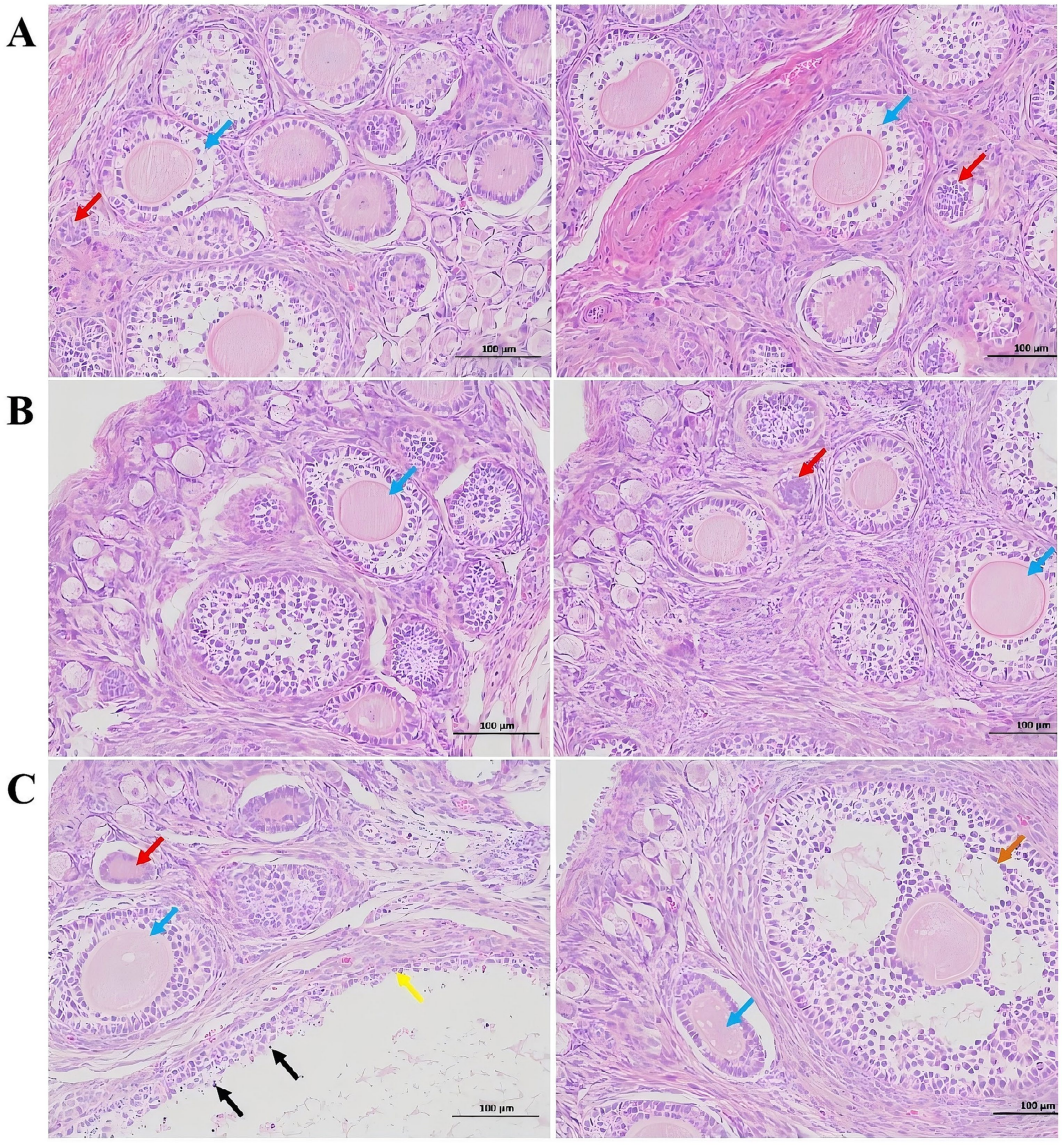
Effect of zearalenone-contaminated diet on the structure and morphology of the ovarian tissues of meat rabbits. **(A)** (Control group), **(B)** (300 μg/kg ZEA supplementation group), and **(C)** (600 μg/kg ZEA supplementation group) illustrate the ovarian tissues at 200× magnification and scales of 100 μm for the control group and experimental groups, respectively (*n* = 8). In these images, red arrows indicate primordial follicles; blue arrows indicate primary follicles; orange arrows indicate secondary follicles; yellow arrows indicate follicular dilation; and black arrows indicate punctate necrosis of granulosa cells.

In the control group, the ovarian tissue of rabbits exhibited a substantial number of follicles at various developmental stages, including primordial and primary follicles (Figure 1). The follicles displayed regular morphology, with no evidence of interstitial proliferation, necrosis, or inflammatory cell infiltration (A). Compared to the control group, no significant pathological changes or follicular changes were observed in the 300 μg/kg ZEA supplementation group (B). However, in the 600 μg/kg ZEA supplementation group, the follicles of meat rabbits exhibited dilation, thinning of the granulosa layer, punctate necrosis of granulosa cells, and shrinkage, deep staining, and fragmentation of granulosa cell nuclei compared to the control group (C).

### 3.3 Serum indicators

#### 3.3.1 Serum reproductive hormone indicators

Compared to the control group, the blood levels of GnRH and E2 were significantly elevated in meat rabbits from the 600 μg/kg ZEA supplementation group (*p* < 0.05) (Table 4). Additionally, the blood LH levels in the 600 μg/kg ZEA supplementation group were significantly higher than those in both the control group and the 300 μg/kg ZEA supplementation group (*p* < 0.05).

**Table 4:** Effects of zearalenone-contaminated diet on serum reproductive hormone levels in meat rabbits.

Items	Control group	300 μg/kg ZEA group	600 μg/kg ZEA group	SEM	*p*-value
GnRH, ng/L	62.66^a^	65.40^ab^	73.07^b^	1.816	0.045
FSH, U/L	17.38	18.53	20.46	0.846	0.338
LH, ng/L	21.54^a^	21.09^a^	28.11^b^	1.326	0.046
E2, ng/L	39.08^a^	45.28^ab^	50.67^b^	2.098	0.032
P, pmol/L	917.69	1018.24	952.50	22.584	0.186

^a–c^Within a row, means with different superscripts differ significantly (*p* < 0.05). Values are means (*n* = 8). 300 μg/kg ZEA, group, 300 μg/kg zearalenone supplementation group; 600 μg/kg ZEA, group, 600 μg/kg zearalenone supplementation group; SEM, the standard error of the means; GnRH, gonadotropin-releasing hormone; FSH, follicle-stimulating hormone; LH, luteinizing hormone; E2, estradiol; P, progesterone.

#### 3.3.2 Serum antioxidant indicators

Compared to the control group, the activities of TAOC and GSH-Px in the blood of meat rabbits in the 600 μg/kg ZEA supplementation group were significantly reduced, while the MDA level was significantly elevated (*p* < 0.05) (Table 5).

**Table 5:** Effects of zearalenone-contaminated diet on serum antioxidant indicators in meat rabbits.

Items	Control group	300 μg/kg ZEA group	600 μg/kg ZEA group	SEM	*p*-value
TAOC, U/mL	44.27^b^	40.73^ab^	34.53^a^	1.480	0.017
GSH-Px, U/L	183.25^b^	175.17^ab^	161.03^a^	3.651	0.034
SOD, U/mL	22.18	22.69	19.86	0.858	0.374
CAT, U/mL	17.75	16.84	18.36	0.673	0.668
MDA, nmol/L	18.19^a^	21.12^ab^	24.71^b^	0.991	0.019

^a–c^Within a row, means with different superscripts differ significantly (*p* < 0.05). Values are means (*n* = 8). 300 μg/kg ZEA, group, 300 μg/kg zearalenone supplementation group; 600 μg/kg ZEA, group, 600 μg/kg zearalenone supplementation group; SEM, the standard error of the means; TAOC, total antioxidant capacity; GSH-Px, glutathione peroxidase; SOD, superoxide dismutase; CAT, catalase; MDA, malondialdehyde.

#### 3.3.3 Serum inflammatory indicators

Furthermore, the blood IL-1β levels in meat rabbits in the 600 μg/kg ZEA supplementation group were significantly elevated compared to the control group and the 300 μg/kg ZEA supplementation group (*p* < 0.05) (Table 6).

**Table 6:** Effects of zearalenone-contaminated diet on serum inflammation indicators in meat rabbits (pg/mL).

Items	Control group	300 μg/kg ZEA group	600 μg/kg ZEA group	SEM	*p*-value
TNF-α	280.46	272.51	312.18	9.686	0.215
IL-1β	46.06^a^	45.42^a^	52.54^b^	1.315	0.042
IL-4	50.61	46.87	48.59	1.528	0.627
IL-6	162.36	172.96	189.80	6.219	0.197
IL-10	786.58	772.68	741.89	12.498	0.328

^a–c^Within a row, means with different superscripts differ significantly (*p* < 0.05). Values are means (*n* = 8). 300 μg/kg ZEA, group, 300 μg/kg zearalenone supplementation group; 600 μg/kg ZEA, group, 600 μg/kg zearalenone supplementation group; SEM, the standard error of the means; TNF-α, tumor necrosis factor α; IL-1β, interleukin-1β; IL-4, interleukin-4; IL-6, interleukin-6; IL-10, interleukin-10.

### 3.4 Ovarian indicators

#### 3.4.1 Ovarian antioxidant indicators

TAOC levels in the ovaries of meat rabbits in the 600 μg/kg ZEA supplementation group were significantly reduced compared to the control group and 300 μg/kg ZEA supplementation group (*p* < 0.05) (Table 7). In addition, compared to the 300 μg/kg ZEA supplementation group, GSH-Px activity in the ovaries of meat rabbits in the 600 μg/kg ZEA supplementation group was significantly lower, while the MDA level was markedly increased (*p* < 0.05).

**Table 7:** Effects of zearalenone-contaminated diet on ovarian antioxidant indicators in meat rabbits.

Items	Control group	300 μg/kg ZEA group	600 μg/kg ZEA group	SEM	*p*-value
TAOC, U/mg prot	38.51^b^	37.39^b^	33.93^a^	0.796	0.042
GSH-Px, U/mg prot	62.78^ab^	65.60^b^	58.02^a^	1.284	0.043
SOD, U/mg prot	29.41	31.15	28.89	0.621	0.310
MDA, nmol/g prot	33.86^ab^	29.20^a^	46.43^b^	1.194	0.035

^a–c^Within a row, means with different superscripts differ significantly (*p* < 0.05). Values are means (*n* = 8). 300 μg/kg ZEA, group, 300 μg/kg zearalenone supplementation group; 600 μg/kg ZEA, group, 600 μg/kg zearalenone supplementation group; SEM, the standard error of the means; TAOC, total antioxidant capacity; GSH-Px, glutathione peroxidase; SOD, superoxide dismutase; MDA, malondialdehyde.

#### 3.4.2 Ovarian inflammatory indicators

Compared to the control group and the 300 μg/kg ZEA supplementation group, the TNF-α level in the ovaries of meat rabbits was significantly elevated in the 600 μg/kg ZEA supplementation group (*p* < 0.05) (Table 8).

**Table 8:** Effects of zearalenone-contaminated diet on ovarian inflammatory indicators in meat rabbits (pg/mg prot).

Items	Control group	300 μg/kg ZEA group	600 μg/kg ZEA group	SEM	*p*-value
TNF-α	130.58^a^	137.36^a^	145.65^b^	1.950	0.003
IL-1β	31.48	32.86	35.50	0.934	0.209
IL-4	34.23	30.75	33.51	0.722	0.114
IL-6	93.96	103.04	95.84	0.190	0.107
IL-10	330.09	346.48	308.08	9.460	0.260

^a–c^Within a row, means with different superscripts differ significantly (*p* < 0.05). Values are means (*n* = 8). 300 μg/kg ZEA, group, 300 μg/kg zearalenone supplementation group; 600 μg/kg ZEA, group, 600 μg/kg zearalenone supplementation group; SEM, the standard error of the means; TNF-α, tumor necrosis factor-α; IL-1β, interleukin-1β; IL-4, interleukin-4; IL-6, interleukin-6; IL-10, interleukin-10.

### 3.5 Expression level of ovarian-related genes

#### 3.5.1 Expression of ovarian *FSHR*, *LHR*, *HSP70*, and antioxidant-related genes

**Figure 2:**
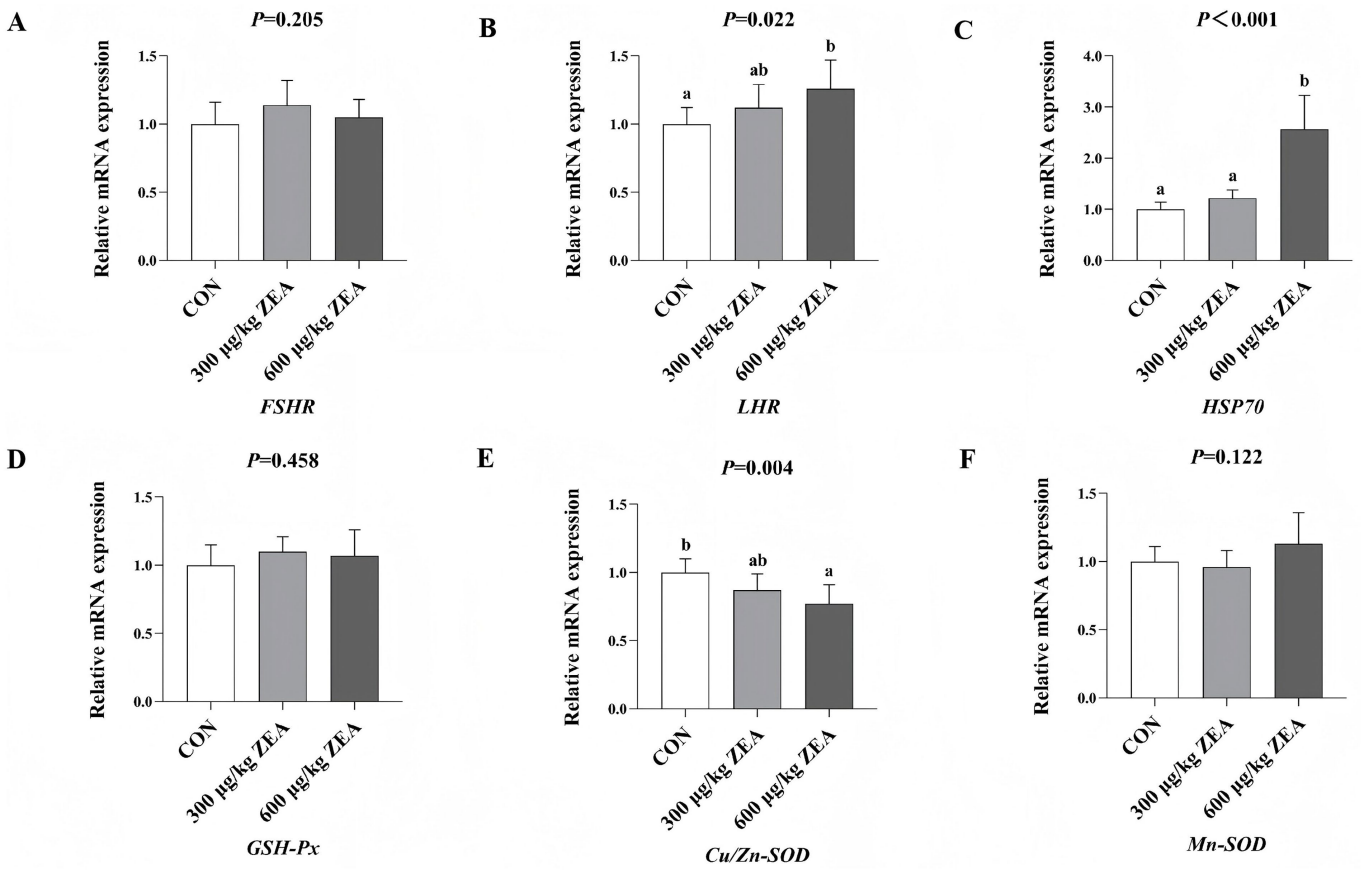
Effects of zearalenone-contaminated diet on the relative expression level of *FSHR*, *LHR*, *HSP70*, and antioxidant-related genes in the ovary of meat rabbits. Bars represent the means ± standard deviation (SD) (*n* = 8). Above the bar, no letter or the same letter means no significant difference (*p* > 0.05), while different letters indicate a significant difference (*p* < 0.05). CON, control group; 300 μg/kg ZEA, 300 μg/kg ZEA supplementation group; 600 μg/kg ZEA, 600 μg/kg ZEA supplementation group; *FSHR*, follicle-stimulating hormone receptor **(A)**; *LHR*, luteinizing hormone receptor **(B)**; *HSP70*, heat shock protein 70 **(C)**; *GSH-Px*, glutathione peroxidase **(D)**; *Cu/Zn-SOD*, copper and zinc superoxide dismutase **(E)**, *Mn-SOD*, manganese superoxide dismutase **(F)**.

As shown in Figure 2, compared to the control group, the abundance of *LHR* mRNA (B) was significantly increased, while *Cu/Zn-SOD* mRNA (E) abundance was significantly decreased in the ovaries of meat rabbits in the 600 μg/kg ZEA supplementation group (*p* < 0.05). Moreover, the abundance of *HSP70* mRNA (C) in the ovaries of meat rabbits in the 600 μg/kg ZEA supplementation group was significantly higher compared to both the control group and the 300 μg/kg ZEA supplementation group (*p* < 0.05).

#### 3.5.2 Expression of ovarian inflammatory response-related genes

**Figure 3:**
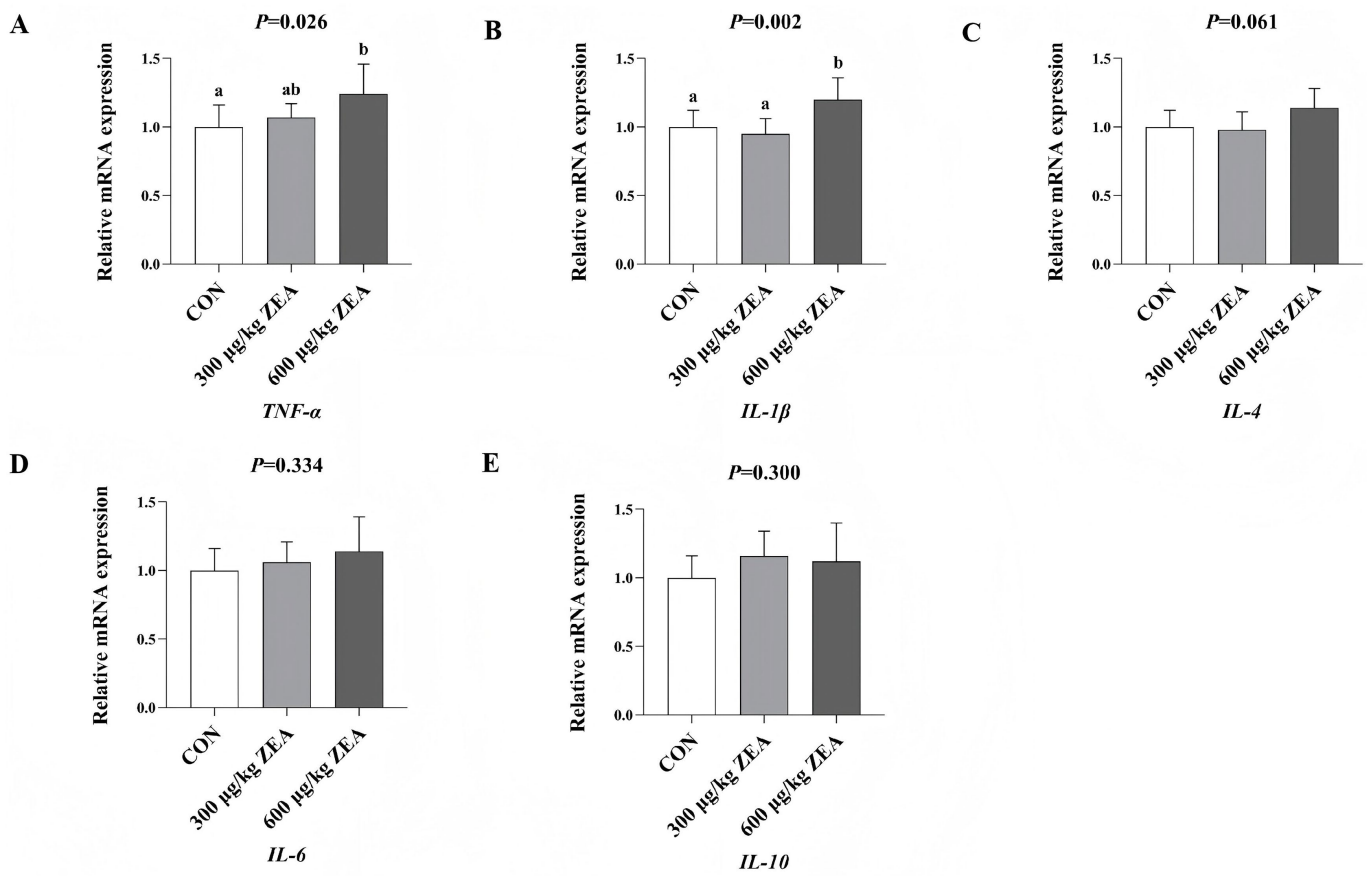
Effects of zearalenone-contaminated diet on the relative expression level of inflammatory response-related genes in the ovary of meat rabbits. Bars represent the means ± standard deviation (SD) (*n* = 8). Above the bar, no letter or the same letter means no significant difference (*p* > 0.05), while different letters indicate a significant difference (*p* < 0.05). CON, control group; 300 μg/kg ZEA, 300 μg/kg ZEA supplementation group; 600 μg/kg ZEA, 600 μg/kg ZEA supplementation group; TNF-α, tumor necrosis factor-α **(A)**; IL-1β, interleukin-1β **(B)**; IL-4, interleukin-4 **(C)**; IL-6, interleukin-6 **(D)**; IL-10, interleukin-10 **(E)**.

Compared to the control group, the abundance of *TNF-α* mRNA (A) in the ovaries of meat rabbits in the 600 μg/kg ZEA supplementation group was significantly increased (*p* < 0.05) (Figure 3). Additionally, the abundance of *IL-1β* mRNA (B) in the ovaries of meat rabbits in the 600 μg/kg ZEA supplementation group was significantly increased compared to both the control group and 300 μg/kg ZEA supplementation group.

## 4 Discussion

ZEA is a highly non-polar, fat-soluble compound that is primarily absorbed in the intestinal tract upon entering the organism. After a series of metabolic reactions, such as monohydroxylation, dehydroreduction, and conjugation in the liver and gallbladder, ZEA’s metabolites and derivatives are distributed to various tissues and organs throughout the body, including the ovaries, uterus, liver, and intestines after reabsorption in the intestinal tract (20–22). It is well known that ZEA can cause oxidative stress and apoptosis in hepatocytes and enterocytes by inducing mitochondrial damage, triggering endoplasmic reticulum stress, and inhibiting antioxidant enzymes. These effects disrupt the organism’s ability to digest, absorb, and metabolize nutrients, ultimately impacting its growth and development (23, 24). However, several studies have reported that ZEA does not significantly affect the growth performance of animals. For example, Wu et al. (9) indicated that feeding rabbits a diet supplemented with 600 μg/kg ZEA for 28 days induced inflammatory response and oxidative stress in the liver but had no significant impact on ADG, ADFI, and F/G. Similarly, Reddy et al. (25) reported that feeding 42-day-old boars a diet supplemented with 800 μg/kg ZEA for 28 days did not significantly affect the ADG, ADFI, and F/G. Peillod et al. (26) found that feeding 84-day-old male ducks a diet supplemented with 500 μg/kg ZEA for 12 days exhibited no significant effect on total feed intake, ADG, and feed conversion ratio. In this experiment, no significant differences in initial body weight, final body weight, ADG, ADFI, and F/G were observed among the groups, indicating that ZEA had no significant effect on the growth performance of meat rabbits, which is partially consistent with the findings reported above. The lack of significant effects may be attributed to the dose of ZEA and the duration of the experiment. In addition, ZEA toxicity mainly targets the reproductive system rather than the digestive system, with reproductive toxicity generally being more severe than growth-related toxicity.

The ovaries serve both reproductive and endocrine functions and are among the major target organs for ZEA-induced reproductive toxicity. Dai et al. (27) investigated the effects of dietary ZEA supplementation in 28-day-old piglets and found that feeding a diet containing 1,040 μg/kg ZEA for 35 days enhanced the autocrine action of growth hormone-releasing peptide, upregulated gene expression in the porcine ovary, and stimulated the development of the ovary and ovarian follicles. Yang et al. (28) supplemented the diets of 25-day-old piglets with 500, 1,000, and 1,500 μg/kg ZEA for 35 days and found that all three doses promoted ovarian development. They further demonstrated that the activation of the estrogen receptors (ERs)/glycogen synthase kinase (GSK-3β)-dependent Wnt inhibitory factor-1 (WIF-1)/β-catenin signaling pathway by ZEA played a critical role in this promotive effect. In this study, the ovarian index of meat rabbits in the 600 μg/kg ZEA supplementation group was significantly elevated, suggesting enhanced ovarian development. This finding partially aligns with the results reported above. Given that there are limited reports on the effects of ZEA on ovarian development in rabbits, further research is needed to determine whether ZEA promotes development through the pathways mentioned above. Inflammatory factors, particularly those involving the production of tumor necrosis factor (TNF), are key contributors to ovarian cell apoptosis in mammals induced by environmental estrogens like ZEA (16). Moreover, ZEA disrupts the balance between oxidative and antioxidant systems, leading to oxidative stress and cellular injury, which are crucial pathways through which ZEA induces ovarian cell apoptosis and damages ovarian tissue (29). In this experiment, pathological changes observed in the ovaries of meat rabbits in the 600 μg/kg ZEA supplementation group, including granulosa cell necrosis and nuclear fragmentation, were associated with ZEA-induced inflammatory response, as evidenced by significant increases in TNF-α levels and gene expression in the ovaries, and oxidative stress, indicated by significantly elevated MDA levels in blood and ovaries.

GnRH, a neurohormone secreted by the hypothalamus, regulates the secretion of gonadotropins by the pituitary gland and has a significant reproduction regulatory effect (30). Yang et al. (31) reported that administering 5 mg/kg of ZEA to 15-day-old rats for 5 days significantly increased the expression levels of GnRH mRNA and protein in the hypothalamus by mediating the kisspeptin-GPR54-GnRH pathway. Additionally, significant increases in serum FSH, LH, and E2 levels, as well as uterine weight, were observed. Similar to the findings of Yang et al. (31) and Kriszt et al. (32) demonstrated that administering 10 mg/kg ZEA to 18-day-old rats for 10 days activated kisspeptin neurons in the periventricular nucleus of the anterior lateral ventricle of the hypothalamus, which in turn stimulated the expression of *GnRH* mRNA, resulting in increased uterine weights and a higher number of follicles in the ovarian sinusoids. In this study, serum GnRH levels were significantly elevated in meat rabbits in the 600 μg/kg ZEA supplementation group. This finding indicates that ZEA exerts a regulatory effect on GnRH in meat rabbits, partially consistent with previous reports; however, the specific underlying regulatory mechanisms remain unidentified. GnRH interacts with its receptors in the pituitary gland via the pituitary portal system, regulating the secretion of FSH and LH (33). In this study, serum LH levels were significantly elevated in meat rabbits in the 600 μg/kg ZEA supplementation group, a response likely associated with the increased GnRH levels. Zheng et al. (33) demonstrated that ZEA and its metabolites inhibit the synthesis and secretion of LH by modulating the GPR30-G-AC/cAMP-PKA-ERK-LHX3-miR-7-FOS pathway in the porcine pituitary. Similarly, Yuan et al. (34) reported a comparable effect on LH levels in 25-week-old laying hens fed diets supplemented with 0.75 mg/kg ZEA for 35 days. Zheng et al. (33) and Yuan et al. (34) reported contrasting results, which may be attributed to species-specific differences in sensitivity to ZEA. However, the precise regulatory mechanisms underlying these differences have not yet been elucidated. A previous study found that LH interacts with the LHR receptor in the ovary to regulate E2 secretion (33). In this study, the expression of *LHR* mRNA and serum E2 levels were significantly elevated in the ovaries of rabbits in the 600 μg/kg ZEA supplementation group. These changes were associated with the increased serum LH levels and the impact of ZEA-induced oxidative stress and inflammatory responses on ovarian endocrine function. The results of this experiment suggest that ZEA exposure may increase the level of GnRH by stimulating the hypothalamus. GnRH can act on the GnRH receptors in the pituitary gland, raising the level of LH, which further acts on the LHR in the ovaries, increasing the level of E2, thereby interfering with the neuroendocrine function of the HPOA.

**Figure 4:**
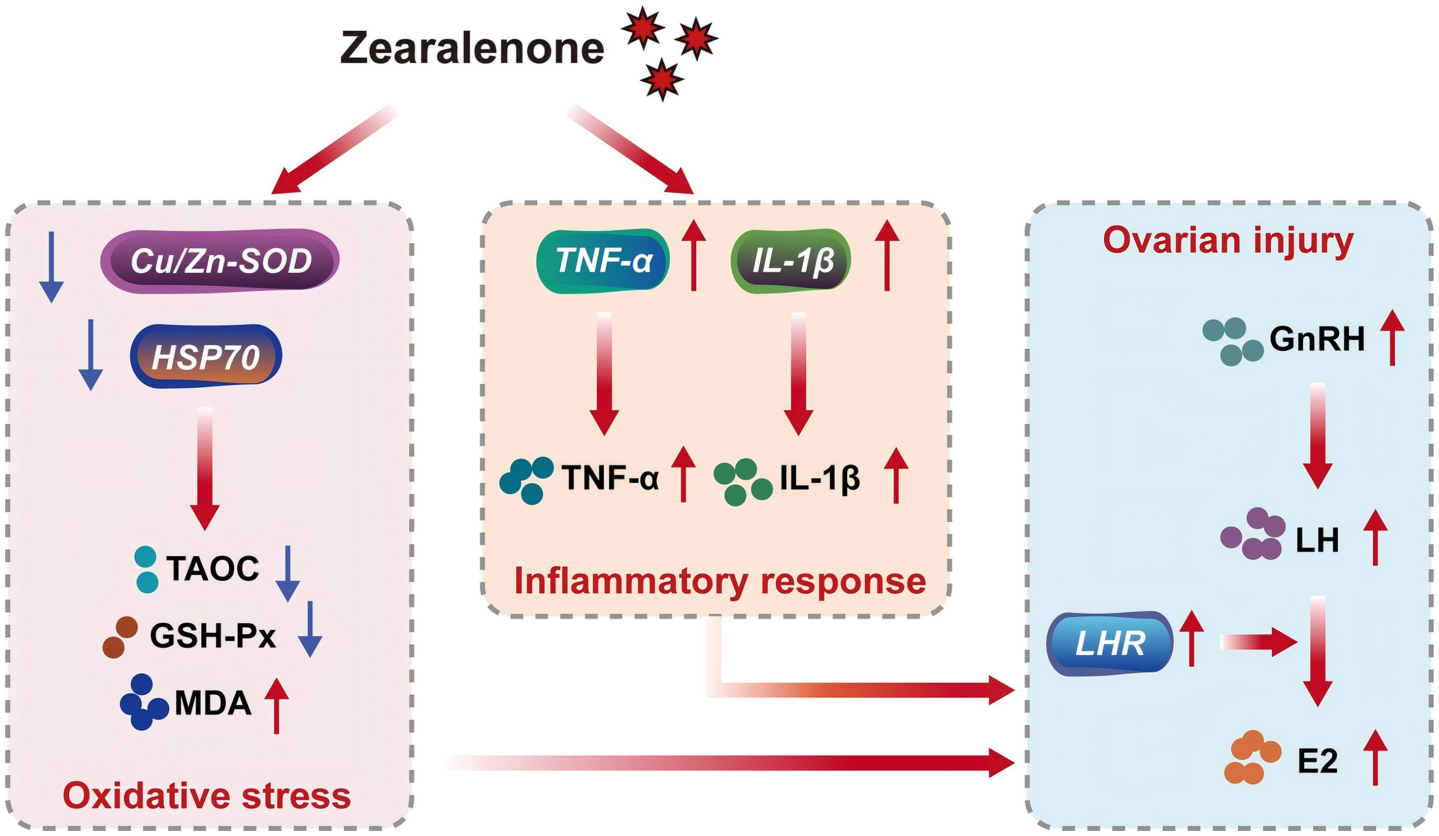
Toxicity and mechanism of zearalenone exposure in meat rabbits. *Cu/Zn-SOD*, copper and zinc superoxide dismutase; *HSP70*, heat shock protein 70; TAOC, total antioxidant capacity; GSH-Px, glutathione peroxidase; MDA, malondialdehyde; TNF-α, tumor necrosis factor-α; IL-1β, interleukin-1β; GnRH, gonadotropin-releasing hormone; LH, luteinizing hormone; *LHR*, luteinizing hormone receptor; E2, estradiol.

ZEA disrupts the balance between oxidative and antioxidant systems in the body, leading to oxidative stress and tissue injury, which contribute to various toxic effects (33, 35–37). This study found that supplementation with 600 μg/kg ZEA significantly reduced TAOC in rabbit ovaries by downregulating the expression of the antioxidant enzyme regulatory gene *Cu/Zn-SOD* mRNA. Meanwhile, the expression of *HSP70* mRNA and serum MDA levels were significantly elevated in the ovaries of rabbits supplemented with ZEA, suggesting that ZEA impacts the antioxidant system of meat rabbits by regulating the expression of antioxidant enzyme-related genes and the activity of antioxidant enzymes, thereby inducing oxidative stress to a certain extent. According to Wu et al. (9), ZEA inhibits SOD and GSH-Px activities in rabbits through the Nrf2/ARE signaling pathway, leading to increased MDA levels and consequent oxidative stress and injury (9). These findings are consistent with some of the results observed in this experiment. Oxidative stress is closely linked to inflammatory responses. ZEA and its metabolites can stimulate electron leakage from the respiratory chain, leading to an increase in free radicals. Subsequently, excessive free radicals can trigger an inflammatory response through the modal or non-modal receptor pathways (38). For example, ZEA can activate ROS-mediated NLRP3 inflammatory vesicles, which in turn promote the activation of the inflammatory cytokine IL-1β via cysteine-dependent aspartic protein hydrolase 1 (39). In this study, supplementation with 600 μg/kg ZEA significantly elevated the TNF-α level by increasing the abundance of *TNF-α* mRNA in the rabbit ovary. Furthermore, both the expression of *IL-1β* mRNA in the ovary and serum IL-1β levels were significantly increased, suggesting the induction of an inflammatory response. These findings align with those reported by Wu et al. (9), who observed similar effects following the addition of ZEA to rabbit diets. The oxidative stress and inflammatory response induced by ZEA in this study were directly linked to ovarian damage and dysfunction. Based on the data from this study, we summarized the toxic effects and mechanisms of zearalenone exposure on rabbits in the form of a schematic diagram (Figure 4).

## 5 Conclusion

In conclusion, supplementation with 600 μg/kg ZEA induces oxidative stress and inflammatory response by modulating the abundance of *Cu/Zn-SOD* mRNA, *TNF-α* mRNA, and *IL-1β* mRNA in the ovaries of meat rabbits. This disruption affects ovarian development, leading to ovarian damage and abnormal estradiol secretion. In contrast, 300 μg/kg ZEA did not have a significant effect on ovarian development or secretion in rabbits.

## Data Availability

The original contributions presented in the study are included in the article/supplementary material, further inquiries can be directed to the corresponding author.
